# A Review on Rail Defect Detection Systems Based on Wireless Sensors

**DOI:** 10.3390/s22176409

**Published:** 2022-08-25

**Authors:** Yuliang Zhao, Zhiqiang Liu, Dong Yi, Xiaodong Yu, Xiaopeng Sha, Lianjiang Li, Hui Sun, Zhikun Zhan, Wen Jung Li

**Affiliations:** 1 School of Control Engineering, Northeastern University at Qinhuangdao, Qinhuangdao 066004, China; 2 Department of Mechanical Engineering, City University of Hong Kong, Hong Kong SAR, China; 3 School of Electrical Engineering, Yanshan University at Qinhuangdao, Qinhuangdao 066104, China

**Keywords:** rail defects detection, wireless sensing system, railway sensors

## Abstract

Small defects on the rails develop fast under the continuous load of passing trains, and this may lead to train derailment and other disasters. In recent years, many types of wireless sensor systems have been developed for rail defect detection. However, there has been a lack of comprehensive reviews on the working principles, functions, and trade-offs of these wireless sensor systems. Therefore, we provide in this paper a systematic review of recent studies on wireless sensor-based rail defect detection systems from three different perspectives: sensing principles, wireless networks, and power supply. We analyzed and compared six sensing methods to discuss their detection accuracy, detectable types of defects, and their detection efficiency. For wireless networks, we analyzed and compared their application scenarios, the advantages and disadvantages of different network topologies, and the capabilities of different transmission media. From the perspective of power supply, we analyzed and compared different power supply modules in terms of installation and energy harvesting methods, and the amount of energy they can supply. Finally, we offered three suggestions that may inspire the future development of wireless sensor-based rail defect detection systems.

## 1. Introduction

During rail service, defects are produced due to material degradation, wheel–rail stress, thermal stress, residual stress [1], and other reasons. If small defects are not discovered and repaired in time, they will be aggravated [2] and in turn cause rail breakage [3] and even serious accidents such as train derailment [4]. Main railway track defects include surface defects, inner defects [5], and component (fastener) defects [6]. With the continuous increase in the railway transportation speed, density, and load [7] there has also been an increase in accidents caused by rail defects. For example, in a mere 10 days in August 2017, four train derailments occurred in India, causing very serious losses [8]. Therefore, the detection of rail defects and the life cycle management [9] of rails has become extremely important. There are also research works focusing on the other part of the railways. For example, Kaewunruen et al. [10] carried out a related study and investigation on the stress of railway sleepers. Setsobhonkul et al. [11] assessed the life cycle of railway bridge transitions exposed to extreme climatic events. Melo et al. showed that the interaction of defects between different parts of the rail can cause different severe consequences, and investigated the related methods for predicting the deterioration of the rail [12].

In the early days, the detection of railway track defects mainly relied on manual detection. Manual inspection is performed by well-trained inspectors who regularly walk along the railway line to identify rail defects. However, manual inspection is inefficient and costly and sometimes even threatens the safety of inspectors [13]. Since the world’s first railway ultrasonic inspection vehicle was put into use in 1959, manual inspection methods have been gradually replaced by large inspection vehicles. For example, in 2004, E. Deutschl et al. [14] designed a vision-based rail surface defect detection system which can automatically detect rail defects. Large-scale rail defect detection vehicles mainly use defects detection devices on the train bogies to detect rail defects [15]. The inspections are conducted once every few months but are not for real-time rail status monitoring [16]. This method occupies the rail, resulting in the inability to transport passengers or cargo during the inspection. Furthermore, neither manual detection nor defect detection vehicles can detect rail damage at the first time when an accident (e.g., derailment and rail breakage) occurs. This means that it is not possible to promptly locate the injury and fix it before damage is caused, and this cannot meet the daily inspection needs of modern railways, especially for high-speed railways [17]. M. Vohra et al. [18] invented a robot-based infrared sensor rail defects detection system. However, the robot-based detection systems are still unable to achieve real-time detection since they usually have larger locomotion ranges than detection ranges. The development of wireless communication and self-organizing networks (GSM, ZigBee ad hoc networks, etc.) enables the information collected by sensing devices to be transmitted to terminal in real time and with good realiabitly [19]. This makes the wireless sensor network a perfect option for the real-time detection of rail defects. E. Aboelela et al. [20] established a wireless sensor network model for railway safety, which laid the foundation for the application of wireless networks in railway track detection. After that, a lot of works [2,21,22,23,24] on the wireless sensor-based rail defect detection systems (WSRDDS) emerged to research the availability of using different sensing methods, wireless commnication, power supply, and data processing in this area. However, there is no systmatic review covering all related aspects of the WSRDDS. From the perspective of data acquisition of the WSRDDS, we focus on the key elements, inlcluding sensing methods, wireless communication, and power supply, in this paper to give an overview.

**Figure 1:**
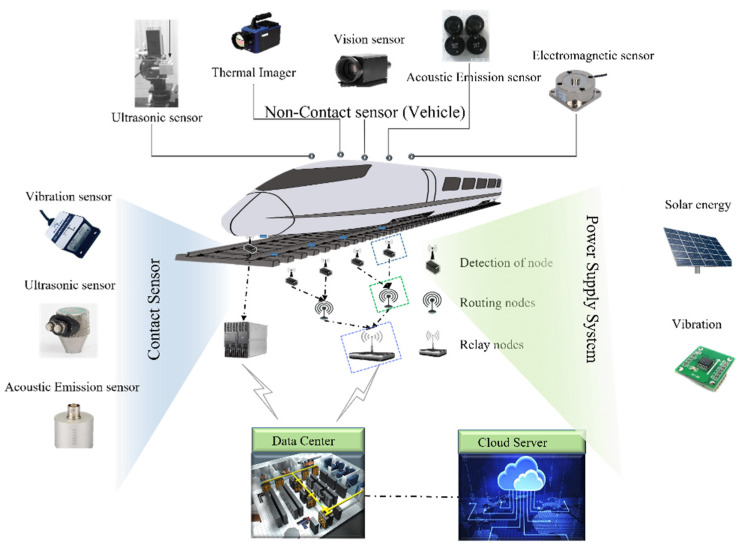
A wireless sensor-based rail defect detection system.

For a WSRDDS, the sensor is the core and should be to be considered first. Different defects require different types of sensors for detecting. To have a long lifetime, a regenerative power supply is required for the wireless sensor. Furthermore, a reliable sensor network architecture should be designed for the data transmission of sensor readings. The main components of the whole system are shown in Figure 1. With the rapid development of high-speed railways, our requirements for the maintenance of rail infrastructure status are increasingly demanded. How to detect rail defects comprehensively, reliably, and in real-time has become extremely important.

## 2. Sensing Method

Since the first rail ultrasonic inspection vehicle was put into use in 1953, various inspection methods have gradually been proposed for rail defect detection [25]. These detection technologies can be summarized as contact detection and non-contact detection based on whether there is physical contact between sensor and rail. Contact sensor detection technology includes: vibration [26], ultrasonic [27,28], and acoustic emission technology [29]. Non-contact sensor detection technology includes: ultrasonic [30], thermal imaging [31], vision [32], electromagnetic wave diffusion [33], etc. In the contact detection methods, the detection sensors are usually installed on the abdomen of the rail [34]. For the non-contact detection methods, the detection sensors are often installed on a large rail detection vehicle [35] or a smart car [36] to detect rail defects. The selection of sensors is highly dependent on the defect types. For example, visual inspection is suitable for detecting rail surface defects, ultrasonic and electromagnetic wave diffusion are suitable for detecting internal rail defects, and thermal imaging is suitable for detecting rail subsurface defects. This section selects a variety of typical sensing methods for the introduction of the sensing mechanism and detectable defect types of these methods. At the end of this section, summarizes the main differences between these methods are summarized.

### 2.1. Vibration

When the train passes the railroad track, it causes vibration of the railroad track [37,38]. There is a significant difference in vibration signals between healthy rails and defective rails. Defective rails have flatter peaks and troughs in the vibration acceleration signal compared to healthy rails [39]. Q. Wei et al. [16] showed that the instantaneous energy distribution is an effective defect feature. For example, among three defects (rail corrugation, ail head sag, and rail surface stripping) the intra-class cross-correlation coefficient of the instantaneous energy distribution is greater than 0.7, while the inter-class ross-correlation coefficient is below 0.45. Therefore, the vibration signal characteristics of different types of rail defects can be extracted through multiple experiments. Finally, classification algorithms can be used to identify and classify rail defects based on these features. M. Sun et al. [40] applied the sequential backward selection (SBS) method to select important feature parameters, and the support vector machine method to recognize and classify the rail defects. This study compares the accuracy of classification before and after using the SBS method and proves that optimizing the parameter set can improve the accuracy of the classification.

MEMS accelerometers are widely used in rail detection due to their small size, low price, and high accuracy [41]. M. David et al. [42] compared MEMS sensors with geophones in 2016. The results prove that MEMS sensors are suitable for track defect detection. Z. Zhan et al. [5] developed a wireless sensor system for rail fastener detection, which can reliably identify fasteners with a looseness coefficient greater than 60%. In addition, strain gauges can also be used to detect missing or broken fasteners. J. J. Zhao et al. [43] demonstrated a linear relationship between the strain voltage and tightness of fasteners, finding that the tighter the fastener, the smaller the strain voltage. We summarized the existing techniques in the literature shown in Table 1.

### 2.2. Acoustic Emission

**Figure 2:**
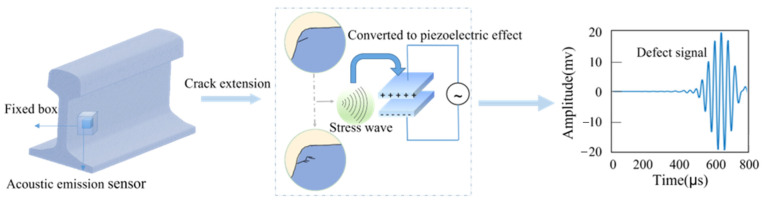
AE sensor detection.

Different from other detection methods, the acoustic emission (AE) method is suited to investigate the dynamic behavior of materials and structures [7]. The dynamic expansion process of rail defects releases transient elastic waves. The acoustic emission (AE) sensor method works based on this phenomenon [45] (as shown in Figure 2). It is more sensitive to the forming and expanding of defect but less influenced by the structural geometry. Furthermore, this method can achieve a detection range as far as 30 m [46]. This method can estimate the dynamic characteristics of defects and is an ideal choice for online continuous monitoring [43]. This method can detect railhead defects, inner defects, welding defects, and surface defects.

H. Jian et al. [47] demonstrated that the acoustic emission frequencies of defective rails are mainly located in the 100–150 KHz and 150–200 KHz frequency bands, and a small part is located in the 380–430 kHz frequency band. In 2013, A. G. Kostryzhev et al. [48] found that the spectral characteristics of the acoustic emission signal depend on the extended mode of the defect. That is, long duration and low-frequency signals come from ductile fractures; short duration and high-frequency signals come from brittle fractures. In 2015, the K.S.C. Kuang team of the Department of Civil and Environmental Engineering of the National University of Singapore [46] found that the railhead side is the best location for inspection. At the same time, the research team used the wavelet transform-based modal analysis location (WTMAL) method to locate defects. The error is less than ±0.30 m in a high-noise environment, and the average working range reaches 30.0 m. However, the defect acoustic emission signal is often interfered with by strong noise. To solve this problem, X. Zhang et al. [29] presented a joint optimization method based on long short-term memory (LSTM) network and k-means clustering to cluster noise signals, and the results showed that most of the noise signals can be reduced. To suppress the influence of noise and ensure proper time resolution, the research team further studied the characteristic frequency of the time window for defect detection [43]. This research has greatly promoted the application of AE sensors in the detection of rail defects. Based on the AE sensor, the dynamic expansion process of the inner defects of the rail can be detected in real-time. However, this method is susceptible to interference from external sound waves (trains and nature). We summarized the existing techniques in the literature shown in Table 2.

### 2.3. Ultrasonic

**Figure 3:**
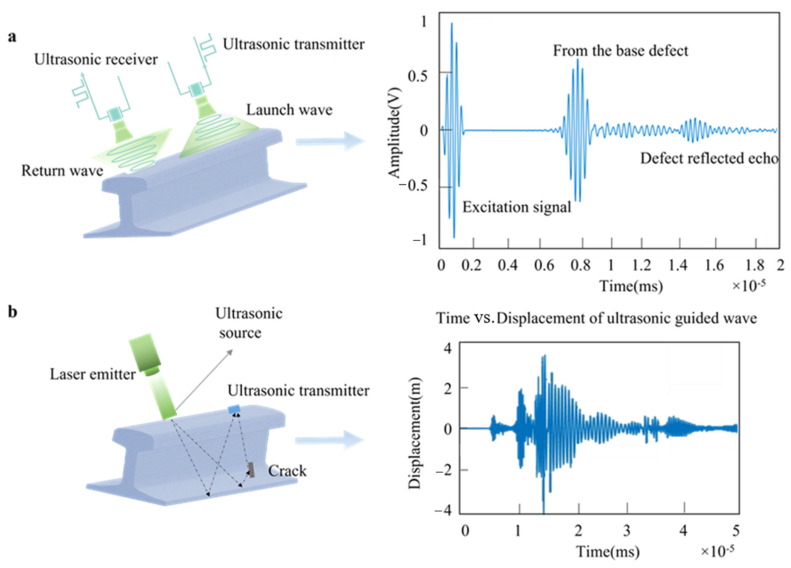
(**a**) Piezoelectric transducer excitation. (**b**) Laser excitation.

The ultrasonic sensor detects the rail defect by analyzing the sound waves reflected from the rail [50]. The prerequisite for the use of ultrasonic sensors for detection is that sound waves must be excited inside the rail. The excitation can be realized by either piezoelectric elements (as shown in Figure 3a) or by lasers (as shown in Figure 3b) and so on. This method has a high detection rate for the inside of the rail (particularly in the railhead and waist) [7]. In this subsection, we summarize the existing techniques in the literature, shown in Table 3.

The focus angle and focus depth of ordinary ultrasound probes are fixed, so the coverage rate of this method on the guide rail is relatively low [30,51]. To overcome the shortcomings of ordinary ultrasonic probes, Zhang et al. [51] proposed a high-speed phased array ultrasonic testing technology. This technology can generate multi-angle beams and receive defect echo signals from all channels, which greatly improves the detection speed and detection range. C. Ling et al. [52] combined traditional probes with phased array probes to detect defects on 60 kg/m rails, and the detection accuracy can reach 6 mm. Acoustic guided waves can cover the entire rail cross section and have a longer propagation distance [53]. Therefore, the efficiency of ultrasonic guided wave detection of rail defects is much greater than the ultrasonic waves. However, different guided wave modes have different sensitivities to defects in different parts of the rail, which greatly increases the complexity of detection [54]. H. Shi et al. [55] studied the mode of the guided wave propagating in the rail at a frequency of 35 KHZ. The study showed that there are a total of 20 guided wave modes propagating in the rail at this frequency. After experimental verification, it proves that modes 7, 3, and 1 are suitable for detecting defects at the rail head, waist, and seat, respectively. Kaewunruen et al. [56] used ultrasonic measurement technology to achieve accurate drawing of the three-dimensional profiles of the deep-sinking defect of the rail. This has important implications for on-site inspections.

Piezoelectric-based ultrasonic technology is applied in almost all the available studies, but the application of this technology is greatly limited by a variety of shortcomings, such as dependence on acoustic coupling agents and requirements for surface pretreatment of the measured object [57]. The laser-based ultrasonic detection technology can excite various modes of ultrasonic waves inside the rail [27,58]. The method has higher accuracy than conventional ultrasonic in the non-destructive detection of small defects in railway tracks.

The ultrasonic sensor emits ultrasound waves that have strong penetration capability and can detect defects in the head and waist of the rail. Under laser excitation, it can further detect defects near the surface and on the foot of the rail. However, when the detection frequency increases, it may easily lose a lot of defect information, and its accuracy is low in detecting very small cracks. We summarized the existing techniques in the literature shown in Table 3.

### 2.4. Electromagnetic 

The motion-induced eddy current (MIEC) is generated on the surface of the rail by the relative motion between the rail and the detection device under the high-speed electromagnetic non-destructive testing [64]. Therefore, it is possible to determine whether defects are present in the rail by analyzing the changes in the inner and surface magnetic fields of the rail [50]. Electromagnetic wave diffusion detection methods are applied on the rail defects detection based on the change, mainly include magnetic flux leakage (MFL) [65] and eddy-current inspection (ECI) [66].

**Figure 4:**
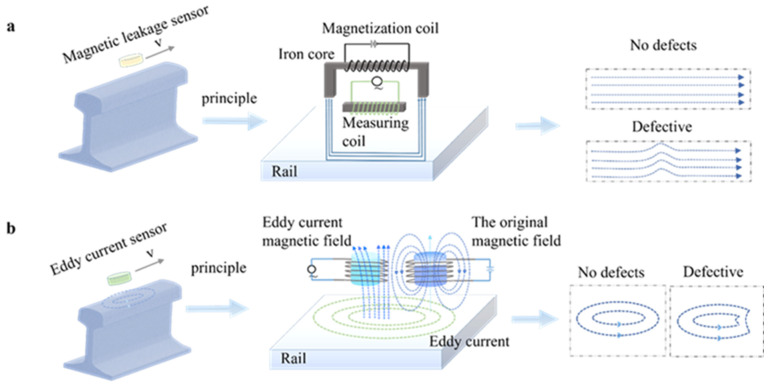
(**a**) Eddy-current detection. (**b**) Magnetic flux leakage detection.

Magnetic flux leakage sensor consists of an excitation source and a detection sensor. Based on the magnetized excitation source, it can be divided into alternating magnetic field, DC magnetic field, and permanent magnet. The sensor first magnetizes the rail under test to saturation through the excitation source [67]. When defects such as cracks or pits appear on the surface of the rail, the evenly distributed lines of magnetic field inside the rail bend to deform and spread outside of the rail (as shown in Figure 4b), forming a leakage magnetic field on the surface of the defect area [68]. For traditional AC magnetic field excitation technology, the excitation signal is usually a single-frequency sinusoidal signal, which cannot accurately extract rail defect information. P. Wang [69] solved this problem by introducing the periodic square wave pulse technology. For high-speed magnetic flux leakage detection, the collected MFL signals often contain complex noise. The increase of detection frequency has an approximately linear relationship with the decrease of the magnetic flux leakage signal. K. Ji et al. [70] proposed an improved adaptive filtering method that can effectively remove noise. L. Yang et al. [71] proposed a high-speed MFL detection technique based on multi-level magnetization to effectively suppress the influence of magnetic after-effects on rail defect detection.

The eddy-current inspection sensor consists of an excitation coil and an induction coil (Figure 4a). Eddy currents cause secondary changes in the strength and distribution of the magnetic field, which leads to changes in the impedance of the detection coil [50]. If no defects are present in the inspection area, the impedance of the detection coil remains constant. If there are defects on and near the surface of the rail, it causes the surface magnetic field to fluctuate and the impedance of the detection coil to change. In this way, rail defects can be detected by analyzing the changes in detection coil impedance. However, many problems are also encountered in high-speed eddy-current testing. For example, the detection signal varies depending on the location of the sensor and the depth of the detection depending on the detection speed. F. Yuan et al. [64] used the DC electromagnetic detection method to study the optimal detection position of rail defects, and the study showed that the optimal detection position is near the inner edge of the excitation coil against the probe movement direction. The team [72] further demonstrated that the PEC detection signal increases with the detection speed, and when the detection speed is constant, the detection signal positively correlates with the defect width and defect depth. 

For electromagnetic detection, the velocity effect can affect the amplitude of the signal, and the signal is subject to greater external interference. Therefore, a well-designed algorithm is needed to offset these effects. Compared with ultrasonic inspection, the electromagnetic inspection can detect near-surface defects. We summarize the existing techniques in the literature, as shown in Table 4.

### 2.5. Thermal Imaging

When an excitation source such as an eddy current is used to excite the rail, a local heating effect is generated inside the rail [75]. The method for analyzing this effect is called thermal imaging detection.

**Figure 5:**
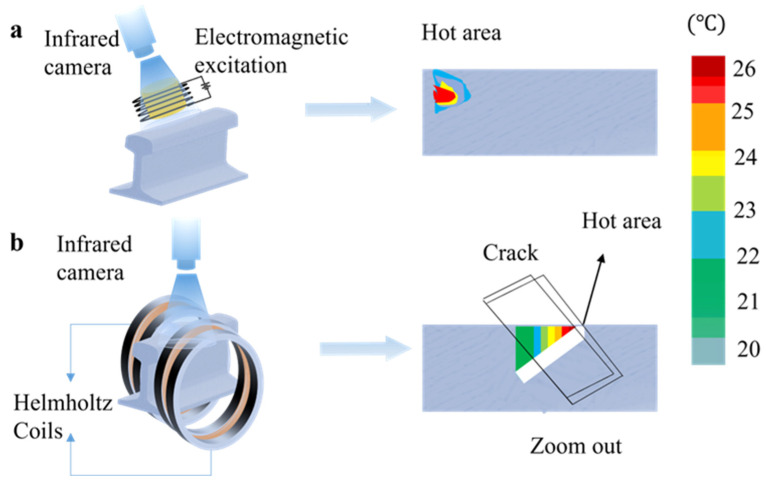
(**a**) Electromagnetic excitation. (**b**) Helmholtz coil excitation.

Pulse thermal imaging based on eddy currents involves two thermal processes in the measurement: Joule heating caused by eddy currents and thermal diffusion inside the material [76,77]. In the heating stage, the presence of surface defects affects the eddy current density distribution and leads to changes in temperature. The geometry of angular defects also affects the temperature difference between the groove edges, which leads to changes in the thermal diffusion mode. Therefore, if the spatial and transient temperature distribution can be obtained, they can be used to detect and characterize the inner defects of the sample [31,78]. This method can accurately detect defects with a width larger than 100 μm [36]. A single-channel blind source separation method for eddy-current pulsed thermography image sequence processing was proposed to extract abnormal patterns and strengthen the comparison of defects [79]. To verify the influence of the sensor’s shape on detection, Y. Wu et al. designed sensors of different shapes for comparison [75]. The results showed that a sensor with a round core structure can only detect partial defects. The arc-shaped and U-shaped sensors can detect almost all defects, and the arc-shaped ones have a higher thermal contrast than the U-shaped ones. However, the excitation of rails by ordinary coils cause angle and instability problems. To solve these problems, J. Peng et al. [76] proposed a Helmholtz coil with a larger and more stable detection range than a linear coil (as shown in Figure 5b) and achieved greatly improved detection efficiency.

There are some other methods for generating thermal effects (as shown in Figure 5a). In 2018, R. Usamentiaga et al. [80] applied optical stimulation to thermally stimulate the rail. The study has shown that when the camera is installed at 1 m from the track, defects of 1 cm^2^ can be detected. 

This technology can detect subsurface defects that cannot be directly discovered on the surface of the rail, greatly improving the ability to detect rail defects. We summarize the existing techniques in the literature, as shown in Table 5.

### 2.6. Visual

**Figure 6:**
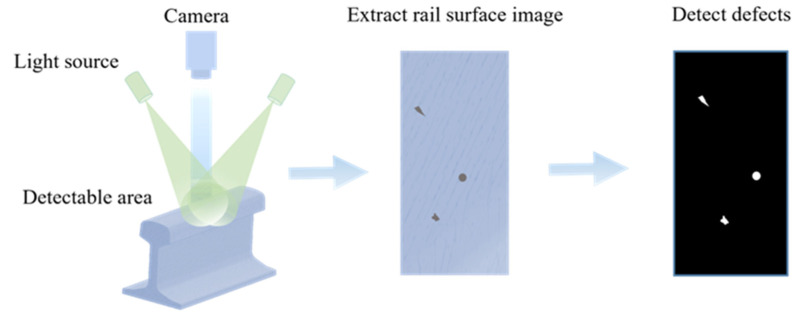
An auxiliary vision system based on a light source.

Visual inspection technology is one of the most important methods in current rail defect detection. An automatic visual inspection system usually consists of a light source, a camera, or other image acquiring devices [85,86] (as shown in Figure 6). The visual inspection system has been widely used in the defect detection of rail facilities along the railway line [50]. According to the visual inspection algorithms, the existing methodologies are categorized into two groups: traditional image processing and deep learning.

The traditional image processing process includes two main steps: extracting effective rail surface images and identifying defects. L. Guo et al. [85] applied Hough transform to extract the image of the effective rail surface, and then applied the improved Sobel algorithm and area filter algorithm to detect rail defects with a minimum area of 0.0068 cm^2^. O. Yaman et al. [87] applied the Otsu segmentation method to extract the image of the rail surface. Next, feature signals are obtained by calculating the variance value through the rail surface image. By analyzing these signals, and combining fuzzy logic to determine the defect type, the success rate can reach 72.05%. Gan et al. [88] proposed a coarse-to-fine extractor for rail defect detection. The method locates the abnormality of rail defects by rough extraction, and then further extracts the defect information. However, the computational complexity of this method is too high.

Due to the extremely complex characteristics of rail surface defects, the use of ordinary image processing techniques cannot achieve good detection results. Z. Liang et al. [89] compared the SegNet (a deep convolutional network architecture for semantic segmentation) algorithm with artificial and automatic threshold segmentation algorithms. The results showed that the accuracy of the deep learning algorithm is 100%, which is much higher than that of ordinary image processing algorithms (77.8% for manual threshold segmentation and 55.6% for automatic threshold segmentation). For the first time, Li et al. [90] combined the U-Net graph segmentation network with the saliency cues method of damage location and applied on the damage detection of high-speed railway rails, with an accuracy rate of 99.76%. L. Zhuang et al. [91] proposed a cascading rail surface flaw identifier. The method detects the presence of defect based on DenseNet-169, and then performs defect classification for the defective rails with a feature joint learning module (FJLM) and a feature reduction module (FRM). 

Visual inspection can effectively detect the surface defects of the rail. However, it does not provide any information about the inner defects of the rails. We summarize the existing techniques in the literature, as shown in Table 6.

### 2.7. Other Detection Methods

There are other rail defect detection methods such as structured light detection, fiber grating detection, and infrared detection.

Q. Mao et al. [94] proposed a fastener detection method based on a structured light sensor. They used a decision tree classifier to classify fastener defects and achieved an overall accuracy no less than 99.8%, indicating that this method can offer a promising way to detect fasteners. Compared with two-dimensional vision, the structured light sensor can obtain a three-dimensional point cloud of fasteners, thereby obtaining more detailed fastener information.

A laser-based non-contact sensor can be an effective tool for detecting rail defects. Generally, the sensor consists of two infrared modules: a transmitter and a receiver. The transmitter emits infrared rays, and the receiver receives the pulses reflected from the rails. By analyzing the time when the pulses are reflected from the rails, the geometric parameters of the rails can be tracked, and a high-resolution map with three-dimensional objects can be generated. This method is effective in detecting surface and welding defects on the rail [18,95].

### 2.8. Technology Comparison

The vibration sensors based on MEMS technology have been widely used. This type of sensor features high accuracy, low price, small size, and convenient installation while having the ability to detect various rail defects through data analysis. The detection accuracy of visual inspection technology is high, but this method can only detect the surface defects. The ultrasonic-based detection technology can detect the inner defects of the rail, but the detection depth is not less than 5 mm [65], which cannot be used for defect detection on the near-surface (≤5 mm) of the rail. Magnetic flux leakage and eddy-current detection technology have high detection accuracy for near-surface defects of rails. The above methods can only detect the static defects of the rails. The acoustic emission detection method is suitable for studying the dynamic expansion process of rail defects. Therefore, the combination of multiple methods can realize the simultaneous monitoring of multiple different defects. In Table 7, a horizontal comparison is provided to distinguish between the different methods.

## 3. Wireless Transmission

In the rail defect detection system, especially in the contact detection technology, many sensors need to be installed on the rail. If wired transmission is used, too many lines need to be placed for data transmission. The rapid advance in wireless communication and self-organizing networks (GSM, ZigBee, others) makes it more reliable and convenient to wirelessly transmit information to terminals [19]. Therefore, reliable wireless transmission is more suitable for rail defect detection than wired transmission [101]. E. Aboelela et al. [20] established a wireless sensor network system for monitoring railway safety which has laid the foundation for the application of wireless networks in railway detection. In the last 10 years, wireless sensor networks have gradually replaced wired monitoring along railway lines. This changes provide enormous convenience for real-time monitoring of railway facilities [101]. Because wireless transmission networks are responsible for data exchange between wireless sensors and terminals, they must be carefully designed to prevent transmission errors, delays, network interruptions, and data loss or damage [19].

### 3.1. Transmission Node Settings

**Figure 7:**
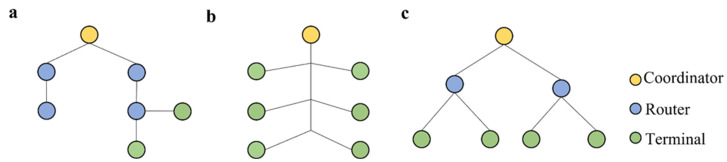
Network topology. (**a**) Tree topology. (**b**) Line topology. (**c**) Star topology.

A good network topology can reduce communication interference, extend the network’s service life, and improve communication efficiency [24]. In a wireless sensor system for rail defect detection, the nodes are usually set up in the following three ways, as illustrated in Figure 7 and compared in Table 8. Each method contains three nodes: the terminal node, routing node, and coordinating node. The terminal node collects data, and the routing node can forward information and assist the coordinator in maintaining the network. The coordinating node is the central hub of the entire network for transmission data.

### 3.2. Transmission Media

In rail wireless sensor systems, the transmission from node to node and from node to base station is usually a short distance, so the wireless communication can be achieved by various technologies such as Bluetooth, Wi-Fi, and ZigBee. In 2007, Aw et al. [104] developed a method that uses Bluetooth to transmit rail detection information. This method, however, has become obsolete due to its weak anti-interference ability and short transmission distance [105]. Zigbee offers limited bandwidth when used for rail condition monitoring [106]. To overcome this disadvantage, M. Tolani et al. [23] designed a two-layer transmission network composed of power-efficient ZigBee nodes as the first layer and bandwidth-efficient WLAN as the second layer. In recent years, Global System for Mobile Communications (GSM) [107] has emerged as a powerful tool for mobile communications [108] and has been used in wireless sensor systems for rail defect detection. Its main advantages are low costs and global availability. Jiaying et al. [102] applied GSM technology to the detection of the environment surrounding the rail, and the results showed that it offers good transmission performance.

### 3.3. Information Transmission

Sustainable running is an essential goal in the design of a wireless sensor network. Therefore, it is important to minimize the energy consumption of the system [109], which can be realized by two main methods: optimizing the transmission protocol and optimizing the hardware design.

In the wireless transmission media access control (MAC) protocol, the energy consumption mainly comes from collision, eavesdropping, and idle monitoring [110]. The schedule-based protocol is collision-free, thus reducing energy waste due to collisions. However, they lack adaptability and scalability to adapt to changes in node density or traffic load. Contention-based protocols have good scalability but cannot avoid wasting energy due to collisions, overhearing, and idle listening [111]. Energy consumption can be reduced by filtering useless data and reducing idle listening time. GM Shafiullah et al. [112] proposed a new protocol named E-BMA. The protocol minimizes the idle time during competition and can achieve improved energy efficiency at low and medium traffic. A. Philipose et al. [113] proposed an improved media access control (MAC) protocol. In this protocol, each node is awakened only when it needs to work, which reduces the energy consumption of sensor nodes.

Another method is to reduce energy consumption by optimizing sensor hardware. This method adopts a sleep strategy when the system is not working, so as to minimize the energy consumption of the system. M. F. Islam et al. [2] proposed a lazy pole strategy, in which data is sent only when the vibration sensed by the sensor node is different from a pre-defined pattern. H. Zhang et al. [114] adopted a synchronous sleep and wake-up strategy to make idle nodes to sleep and shut down most hardware to greatly reduce energy consumption.

Therefore, for future designs of wireless sensor networks for rail defect detection systems, these two methods can be combined to minimize energy consumption.

## 4. Power Supply

**Figure 8:**
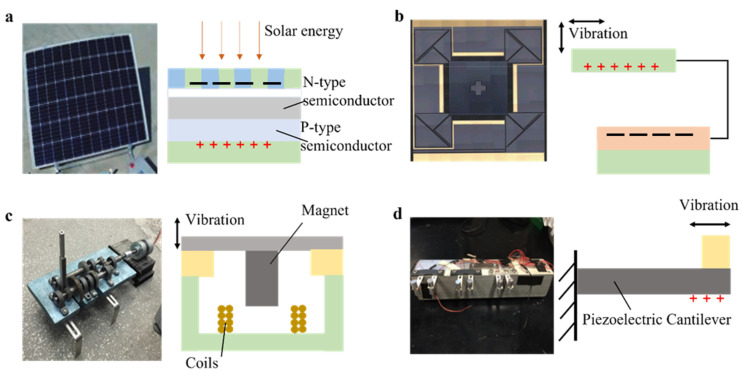
Principles of power generation. (**a**) Principle of solar energy harvester [34]. (**b**) Principle of electrostatic harvester [118]. (**c**) Principle of electromagnetic harvester [119]. (**d**) Principle of piezoelectric energy harvester [120].

It is extremely important to make a wireless sensor self-powered to reduce the cost of maintenance and have a long lifetime. The energy generated by the surrounding environment such as rail vibration [115], solar energy [116], and other types can be stored in a rechargeable battery [117] for powering the wireless sensing detection system. The power generation methods of the rail wireless sensor detection system at present are shown in Figure 8.

### 4.1. Solar

Solar power generation methods include photovoltaic power generation (light energy is converted into electrical energy) and thermal power generation (thermal energy is converted into electrical energy) [121]. Photovoltaic power generation can achieve a power density as high as 10–15 mW/cm^2^ [116], which a enough to power wireless sensors. Solar thermal power generation is a technology that uses solar concentrators to convert solar radiant energy into thermal energy and then into electrical energy. However, solar thermal power generators cannot be used on a large scale in wireless sensors as they are highly susceptible to weather conditions and other environmental impacts, and it is difficult to find a suitable location to install them.

### 4.2. Vibration

Vibration power generation converts the kinetic energy of rail vibration into electrical energy. Typical vibration power generators include electromagnetic, piezoelectric, and electrostatic generators [106]. An electromagnetic generator converts the orbital vibration into the relative motion between the permanent magnet and the coil [119] and converts it further into extremely low-frequency electrical energy [122]. For the first time, X. Zhang et al. [119] applied supercapacitors to vibration energy harvesting systems. The system amplifies the small vibrations of the track and store energy from rapidly changing transient currents. For piezoelectric power generation, when pressure is applied to a piezoelectric material, a potential difference is generated on the surface of the piezoelectric material [123,124]. J. Wang et al. [125] studied a theoretical model of using the patch and stacked piezoelectric transducers to collect piezoelectric energy from railway systems. The electrostatic generator needs to be driven by an external voltage, so it features high output impedance and high voltage and is not readily applicable for sensing devices. Table 9 summarizes the main differences of the related studies. These technologies focus on converting environmental energy into electrical energy [120].

A solar power generator is highly dependent on external conditions. It requires stable light conditions, which make it difficult to find an appropriate location for sensor installation. A vibration-based piezoelectric energy collector has the advantages of a simple structure and a small size. However, this method requires a large vibration amplitude of the rail. An electromagnetic energy harvester is much less demanding on the amplitude of rail vibration, but it is susceptible to external electromagnetic interference.

## 5. Summary and Future Work

This paper reviews the existing conventional rail detection technologies such as vibration, ultrasonic, electromagnetic detection, and visual detection and makes comparisons between them and briefly introduces the wireless transmission method and power generation methods for the WSRDDS.

There is still some potential improvement for existing systems. We suggest the following optimizations to make these systems more reliable, intelligent, and powerful in detecting rail defects.

(1) Rail defect feature signals can be extracted to build a complete database of rail defect and fastener defect features. This database can be used to automatically classify rail defects and determine the degree of damage to other track components.
(2) For a single detection technology, it is difficult to detect all the information from the rails. Combining a variety of sensors can achieve all-round and high-precision detection of rail defects.
(3) Building a comprehensive monitoring system for rail defects based on big data management and information mining technology is a good direction for achieving all-round and high-precision detection of rail infrastructure.

## Figures and Tables

**Table 1:** Comparison of vibration testing methods.

Methods	Types of Detected Defects	Algorithm	Results	Comments
MEMS accelerometers	Rail fastener [5]	Finite element method	Reliable identification of fasteners with a looseness factor greater than 60%	Small size, low price, high accuracy
	/ [42]	The high- and low-pass filter	This study proves that MEMS sensors are suitable for rail defect detection.	
	Rail head sag, rail surface stripping, height joint [40].	Peak-finding algorithm	The accuracy rate of the classification of rail defect types can reach 93.8%.	
Strain gauge	Rail fastener [43]	Sequential backward selection	Demonstrated a linear relationship between strain voltage and fastener tightness.	Small size, low price, low accuracy
	Rail fastener [44]	Support vector machines	Demonstrated a linear relationship between strain voltage and fastener tightness.	

**Table 2:** Comparison of acoustic emission methods.

Methods	Types of Detected Defects	Algorithm	Results	Comments
AE	Rail-head defects [46]	Hilbert transform Wavelet transform	The error of the location of rail defects is less than 0.3 m. Detection distance can reach 30 m.	Long detection distance
AE	/[49]	Signal adapted wavelet in the frame of a two-band analysis/synthesis system	The wavelet designed by the proposed method has superior performance in expressing the defect AE signal, and can outperform the most suitable existing wavelet.	The designed wavelet shows good robustness against noise, which has profound meaning for rail defect detection in practical applications.
AE	Rail fatigue defect [48]	Single-hit waveform and power spectrum analysis	High duration, low frequency signals result from ductile fractures. Low duration, high frequency signals result from brittle fractures.	It is demonstrated that the AE signal associated with defect propagation depends on the fracture mode.
AE	Rail defect, small bearing defect, and worse bearing defect [47]	Cepstrum analysis	This study verifies that AE signals can detect bearing/rail defects.	

**Table 3:** Comparison of ultrasonic testing methods.

Methods			Algorithm or Simulation	Types of Detectable Defects	Results	Summarize
Detection Method	Ordinary ultrasound	Multi-angle ultrasonic probe [59]	PCA and LSSVM	Different types of defects in rail head, rail waist and rail foot	Classification recognition accuracy: 92%. Identify seven types of rail defects.	Ordinary ultrasonic waves are usually single-modal at low frequencies, and cannot achieve high-sensitivity omnidirectional detection of all parts of the rail (track surface, underground, and interior).
		Combination of wheeled ultrasonic probes [60]	LSTM-based deep learning model		Average f1-score: 95.5%. Maximum detection speed: 22 m/s.	
	Phased array ultrasonic	Combination of the conventional probe and phased array probe [51]	/	Defects around bolt holes, vertical defects and transverse imperfections in the rail head, waist and foundation area	Ultrasonic beam coverage rate up to 80%	The rails can be inspected more comprehensively and the inspection efficiency is improved. Multiple angles monitoring the same area.
		Phased array with transverse wedge block(railhead), transverse and longitudinal wave probes (rail waist and rail foot) [61]	/	Different types of defects in rail head, rail waist and rail foot	Effectively covers the railhead, rail foot, and rail waist	
		Combination of the conventional probe and phased array probe [52]	/	Different types of defects in rail head, rail waist and rail foot	The detection accuracy can reach 6 mm.	
	Ultrasonic guided wave	High voltage pulse sequences [62]	/	/	Coverage up to 1000 m	The efficiency of ultrasonic guided wave detection of rail defects is much greater than the ultrasonic waves.
		Sine wave modulated by the Hanning window with a frequency of 35 kHz [55]	Phase control and time delay technology.	Rail head, rail waist and rail foot	Enhance expected mode and suppress interference mode. The optimal excitation direction and excitation node of the modes are calculated.	
Excitation source	Laser ultrasonic	High energy laser pulses [58]	Finite element simulations	Rail foot	The best detection position is 300 mm in front of the defect position. The best detection frequency is 20 KHZ.	Can cover the head, web, and foot parts of the rail
		Non-ablative laser source [63]	Analysis of Variance. Monte-Carlo simulations.	Head surface defects, horizontal defects, vertical longitudinal split defects, star defects at colt holes and diagonal defect in waist.	The position of the sensor has a greater impact on detection accuracy. The research results can find the best detection position of the sensor.	
		Hybrid laser/air coupling sensor system [35]	Wavelet transform and outlier analysis.	Surface defects(Transverse defects and alongitudinal defects)	Inner defects and surface defects of the rail can be distinguished.	
		Two staggered beams of laser [27]	Finite element simulations.	Irregular scratches on rail surface	The error is about 0.014%.	
	Electromagnetic ultrasonic	/	Finite element analysis [57]	Rail base	Able to detect common defects in rail bases	No couplant required

**Table 4:** Comparison of electromagnetic testing methods.

Methods		Algorithm or Simulation	Types of Detectable Defects	Research Content and Results
Eddy current	Pulsed eddy current [72]	3D transient model	Different installation positions can detect rail defects in different parts.	The team studied the relationship between the pulsed eddy current detection signal and the velocity of different defect depths and widths.
	Direct current [64]	2D Finite element method	Different installation positions can detect rail defects in different parts.	The optimal detection position is determined.
	AC bridge techniques [73]	Digital lock-in amplifier algorithm	Four typical types of rail defects (transverse defects, compound fissure, crushed head, detail fracture)	The effect of solving the lift-off effect is better.
	Differential eddy-current (EC) sensor system [33]	Low-pass filter Rotation of EC signal (To extract maximum information and have better visualization)	The degree of looseness of fasteners	Can detect fastener features 65 mm above the track The type of missing fixture can be detected by analyzing the characteristics of the fastener.
Magnetic flux leakage	Pulsed magnetic flux leakage [69]	2D transient analysis model under	Vertical and oblique defects	With the sensor array, not only the magnetic field distribution of the defect can be detected, but also the edge effect caused by the magnetic yoke can be eliminated. The introduction of periodic square wave pulses solves the problem that single-frequency sinusoidal signals cannot effectively extract rail defect information.
	Multistage magnetization [71]	Finite element method	Rail inner defects	Magnetic aftereffects are effectively inhibited in high-speed MFL detection.
	Direct current [68]	2D simulation model	Oblique defect and rectangle defect	Analyzed the influence of speed on magnetic flux leakage signal (At high speed, the magnitude of the flux leakage signal is smaller, but more stable.)
	Magnetic flux leakage [70]	Improved adaptive filtering	Different types of defects in rail surface	The noise intensity of the MFL signal is reduced by up to about 80%. The generalization ability of the algorithm is better, and the filtering effect becomes more significant as the speed increases.
	Combination of permanent magnets and yoke [74]	3-D FEM simulations	Different types of defects in rail surface	The MFL signals from the subsurface defect will be more affected by the weakly magnetized regions compared to the surface defect. The increase in speed reduces the magnetization of the rail.

**Table 5:** Comparison of thermal imaging testing methods.

Thermal Stimulation		Algorithm	Types of Detectable Defects	Results	Comments
Eddy current	Eddy-current pulsed thermography [79]	Single-channel blind source separation	Thermal fatigue defects	The method can automatically detect rail defects in both the time and the spatial domains.	The research innovatively discovered the changing process of the mixing vector in the heating and cooling phases.
	Helmholtz coils [76]	Finite element method	Rolling contact fatigue (RCF) defects	Solved the problem that the excitation of ordinary coils on the rails would cause unstable detection areas	This method provides a larger detection area than linear coils.
	Various shapes of sensors [75]	Inverse Fourier transformation (deblurring method)	RCF defects and micro-defect	Verify the detection effect of various shape sensors	The research is helpful to design sensors with better detection performance.
	Easyheat 224 system with induction heater [81]	Normalized difference vegetation index (NDVI)	RCF defects	The proposed method can have a good correction for the emissivity.	Good for correcting ECPT emissivity
Laser	Two halogen lamps [80]	/	Rolled-in material defect	Defects of 1 cm^2^ can be detected.	The study compared multiple methods to enhance the defect signal-to-noise ratio.
Pulsed air-flow thermography [82]		Subtract the first image in the sequence from the last image acquired in the heating sequence when removing the background.	Rail surface defects	The study proved that the pulsed air-flow thermography method used in the experiment is effective for detecting rail defects.	The method needs further improvement.
High-frequency continuous sine-wave current [83]		Metric learning modules	Fatigue defects	The method proposed in this study can not only reduce the influence of interference factors but also expand the feature space distance between defective samples and normal samples.	Using an open set of supervision frameworks, it is easy to add new defect samples. Good anti-interference performance
Apply uniform heat flux for a time [84]		pulse phase thermography (PPT)	Lateral surface defects	After thermal stimulation for the same time, the cooling rate of shallow defects is faster than that of deep defects.	The study proved the feasibility of active infrared thermography for detecting rail defects.

**Table 6:** Comparison of visual inspection methods.

Algorithm		Results	Comments	Summarize
Traditional algorithm	Hough transform and improved Sobel algorithm [85]	Minimum detection area: 0.0068 cm^2^	Fast processing speed Harder to apply to complex situations	Weak generalization ability and low accuracy
	Otsu segmentation and fuzzy logic [87]	The success rate of identifying defect types: 72.05%	Types of defects can be identified.	
	Coarse-to-fine model [88,92]	CTFM outperforms state-of-the-art methods in terms of pixel-level indices and defect-level indices.	Effectively suppress the influence of noise points The proposed computational requires high computational resources.	
Deep learning	SegNet [89]	Detection accuracy:100%	outperform ordinary image processing algorithms	Strong generalization ability and high accuracy
	SCueU-Net [90]	Detection accuracy:99.76%	Overcome the interference of image noise and solve the current problem of low detection efficiency	
	MOLO [93]	This algorithm improves the accuracy 3–5% more than the YOLOv3 algorithm.	Image features are extracted using MobileNetV2 as the backbone network. At the same time, the multi-scale prediction and the loss calculation method of YOLOv3 are used. The network structure is relatively simple, which balances detection accuracy and detection speed.	
	Cascading rail surface flaw identifier [91]	The detection accuracy rate of defect type: 98.2%	Better processing performance for complex scenes Accurately identify multiple types of defects	

**Table 7:** Comparison of rail defect detection methods.

Detection Method			Types of Detectable Defects	Detection Performance		Influence of Environment on Detection Performance
Vibration accelerometer			The degree of looseness of fasteners [5,96] Inner [2] and surface [47] defects of the rail	Can detect the degree of looseness of fasteners [5] Small size, easy installation, wide detection range [42]		Temperatures that are too low will reduce the sensitivity of the sensor.
Ultrasonic	Ordinary ultrasonic [51,52,61]	Conventional probe	Railhead inner defects Rail foot defects Rail waist defects	Single angle and low efficiency	In high-speed inspection systems, rail defects with a depth of less than 4 mm are often undetectable [76].	When the temperature changes, it will affect the speed of the sound wave in the rail, so the localization of the defect will have an impact.
		Phased array probe		Multi-angle detection Better ultrasonic beam coverage Higher efficiency than traditional ultrasonic testing		
	Electromagnetic ultrasonic		Rail inner defects surface defects	High precision No complaint required [57,97]		
	Laser ultrasonic		Rail inner defects [35] Surface defects [98] and subsurface defects [58]	Good penetration ability Can cover the entire track for testing [63]		
AE			Subsurface defects [48]	Suitable for studying the dynamic expansion process of rail defects [48] Acoustic emission signals are easily submerged by high-frequency vehicle speed signals [48].		Other noises will affect the detection results.
Electromagnetic	MFL		Surface and shallow surface defects [69,74]	Highly susceptible to the environment (white noise and power frequency interference in the environment) [71] Easily affected by lift-off [64,74] As the detection rate increases, the depth of detection of rail defects decreases [64]		The temperature will drift the detection results of the eddy-current sensor, and the two are negatively correlated.The increase in temperature will cause the magnetic permeability to decrease.
	ECI		Rail inner defects [99] surface and subsurface defects [64]	Easily affected by lift-off [64]. Can effectively detect subsurface defects.		
Thermal imaging			Subsurface defects [76] and surface defects [82]	It can characterize the shape and size of rail defects [76,80].		Contamination present on the Rail surface will attenuate the signal.
Vision			Missing fastener fixture [100] Surface defects [93]	Can only detect surface defects High detection accuracy Mature detection algorithm Affected by the surface condition (dirt occlusion, others)		Contaminants such as snowflakes and leaves can block rail defects, making visual inspection methods unable to detect rail defects.

**Table 8:** Comparison of network topology.

Network Topology	Advantages	Disadvantages	References
Star topology	Short network delay time Simple structure Easy to maintain	Low line utilization The central node load is too heavy.	[43,102]
Tree topology	Simple structure Easy to maintain Easy to expand	The dependence of each node on the root is too large.	[20,24]
Line topology	Simple structure Low cost Easy to expand	Low reliability Difficulty in fault diagnosis and isolation	[103]

**Table 9:** Comparison of energy harvesters based on vibration principle.

Energy Harvesting Device	Application Conditions	Installation Location	Voltage	Power	Reference
Piezoelectric energy harvester	2.5 mph (the speed of the train) The resistor connected in the PZT0 (a single piezoelectric energy harvester) was 9.9 KΩ	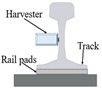	40 V (the maximum voltage)	0.18 mW (the maximum power)	[120]
Magnetic levitation oscillator	105 km/h (the speed of the train) (one-car train)	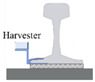	2.3 V (peak–peak output voltage)	/	[106]
Galfenol magnetostictive device	60 km/h (the speed of the train) 60 m (The train is far from the sensor of 60 m.)	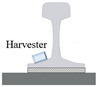	0.15 V (The voltage varies with the distance between the train and the sensor, when the distance is shorter, the voltage is larger, and the longer the distance, the smaller the voltage.)	When the terminal voltage is about 0.56 V, the power is maximum.	[115]
A patch-type piezoelectric transducer	30 m/s (the speed of the train)	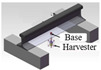	4.82 V (at the beginning of a valid signal)	0.19 mW (at the beginning of a valid signal)	[125]
Drum transducer	0.15 m/s (running speed) 120 kg (the weight of a fully-loaded train)	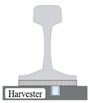	50–70 V (peak open-circuit voltage)	100 mW	[123]
Electromagnetic energy harvesting system	6 mm (amplitude) 1 Hz and 2 Hz (frequencies)	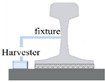	6.45 V (the output peak–peak voltage)	0.0912 J	[119]
Magnetic levitation harvester	low-frequency (3–7 Hz) Rail displacement	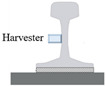	2.32 V (the output peak–peak voltage)	119 mW	[126]

## Data Availability

Not applicable.
